# Time Since Immigration and Ethnicity as Predictors of Physical Activity among Canadian Youth: A Cross-Sectional Study

**DOI:** 10.1371/journal.pone.0089509

**Published:** 2014-02-21

**Authors:** Atif Kukaswadia, William Pickett, Ian Janssen

**Affiliations:** 1 Department of Public Health Sciences, Queen’s University, Kingston, Ontario, Canada; 2 Clinical Research Centre, Kingston General Hospital, Kingston, Ontario, Canada; 3 School of Kinesiology and Health Studies, Queen’s University, Kingston, Ontario, Canada; Old Dominion University, United States of America

## Abstract

**Background:**

Little is known about patterns of physical activity engaged in by youth after they immigrate to a new country. This study aims to investigate relationships between immigrant generation and ethnicity with physical activity, and to determine if the relationship between immigrant generation and physical activity was modified by ethnicity.

**Methods:**

The data sources were Cycle 6 (2009–2010) of the Canadian Health Behaviour in School-Aged Children Study and the 2006 Canada Census of Population. Participants (weighted n = 23,124) were young people from grades 6–10 in 436 schools. Students were asked where they were born, how long ago they moved to Canada, their ethnicity, and how many days a week they accumulated at least 60 minutes of moderate-to-vigorous physical activity (MVPA).

**Results:**

Youth born outside of Canada were less likely to be active than peers born in Canada; 11% vs 15% reported 7 days/week of at least 60 minutes of MVPA (p = .001). MVPA increased with time since immigration. Compared to Canadian-born youth, youth who immigrated within the last 1–2 years were less likely to get sufficient MVPA on 4–6 days/week (odds ratio: 0.66, 95% confidence interval: 0.53–0.82) and 7 days/week (0.62; 0.43–0.89). East and South-East Asian youth were less active, regardless of time since immigration: 4–6 days/week (0.67; 0.58–0.79) and 7 days/week (0.37; 0.29–0.48).

**Conclusion:**

Time since immigration and ethnicity were associated with MVPA among Canadian youth. Mechanisms by which these differences occur need to be uncovered in order to identify barriers to physical activity participation among youth.

## Background

An important determinant of the health of young people is involvement in regular physical activity. [1] It is recommended that youth accumulate at least 60 minutes of moderate-to-vigorous physical activity (MVPA) daily. [2]–[4] In Canada, only 9% of boys and 4% of girls meet these criteria. [5] Gender, age, and socio-economic status are well documented correlates of physical activity. [6], [7] Despite its potential importance, the role of immigration as a determinant of physical activity is understudied. A migrant refers to someone who has moved to a new country. [8] When an individual immigrates to a new country, two different cultures meet and acculturation occurs. [9] Acculturation refers to behavioural and psychological changes that occur as individuals adopt the norms and values of their new or host culture, while balancing these norms with those of their heritage culture. [9] These may manifest as changes in health and health-related behaviours, such as physical activity [10], [11].

Acculturation can manifest in two types of health outcomes: psychological outcomes [12] and behavioural adaptation. [13] Psychological outcomes refer to internal adjustments made, and include emotional and psychological issues such as life satisfaction. [12], [14] Behavioural adaptations are external changes made, and refer to acquiring culturally appropriate skills and knowledge, obtained through interaction with the host culture. [13] These changes can be associated with both positive and negative health outcomes. While youth who do not integrate have lower rates of obesity and higher levels of active transportation, [15], [16] they are also more likely to be in physical fights and have higher levels of alcohol consumption. [16], [17] These differences are especially noticeable when the host culture, i.e. Canada or the US, and the heritage culture, i.e. where the youth and their parents immigrated from, are very different. This is relevant to the US and Canada as immigrants from Asia and the Pacific account for over 40% of all new immigrants, and also in Europe where Asian immigrants are the fourth largest immigrant group, and have different cultural norms and values compared to European peers [18]–[20].

One methodological problem faced by researchers is that acculturation is a multidimensional process that is difficult to measure. Immigrant generation or time since immigration are often used as proxy measures of acculturation, with the assumption that higher immigrant generation or longer time since immigration are associated with increased acculturation. With the exception of one study, [21] studies of the experiences of immigrant youth have concluded that physical activity levels are lower among foreign-born youth compared with those born in the host country [16], [22]–[26] However, this relationship may be confounded by time since immigration. Time since immigration may more accurately capture acculturation as a determinant of health. This has been explored among Canadian adults aged 20 and older in the Canadian Community Health Survey. This study found that recent immigrants (<10 years) are 2.68 (95% CI: 2.54–2.83) times more likely to get no meaningful physical activity compared to non-immigrants, which is far greater than the risk observed in established immigrants (>10 years) (OR: 1.30, 95% CI: 1.26–1.35). [27] However, prior research has focused on changes in adult physical activity over time. Youth are different than adults, and the etiology and determinants of physical activity among youth may be unique. In addition, due to the rapidly growing size of the immigrant youth population, this group warrants specific study.

Previous studies have typically focused on the experiences of a specific ethnic group living within a single geographical area, thus limiting their generalizability. [16], [21]–[24], [26], [28] One study examined a diverse national sample of US youth, and it found that immigrants are less active than those born in the host country. [25] These studies of differences between ethnic groups have limited generalizability to Canada as immigrants to the US and Europe are from different geographical regions than Canadian immigrants, and ethnicity is an important determinant of physical activity. [18]–[20], [29] A Canadian study examined the relationship between immigrant generation and physical activity among youth from low-income, inner city neighbourhoods in Montreal, Quebec, found that youth who had spent less than 25% of their life in Canada were less active. [30] However, this study did not consider ethnicity or interactions between time youth lived in Canada or ethnicity, which could mitigate this relationship. [31] Thus, studies are needed that compare how different ethnicities adapt to new cultures and contexts.

Our aim was to investigate differences in MVPA levels between immigrant youth and their Canadian-born peers. Subanalyses investigated whether this relationship differs by time since immigration and ethnicity. We hypothesize that immigrant youth would have lower MVPA than non-immigrants, and that this differs by ethnicity.

## Methods

### Data Sources

Individual-level data were obtained from Cycle 6 (2010) of the *Canadian Health Behaviour in School-Aged Children* (HBSC) Study. HBSC is an international survey conducted in affiliation with the World Health Organization. It is a self-reported general health survey completed by students in the classroom setting. [32] The 2010 Canadian HBSC collected information from 26,078 youth in grades 6 through 10 in 436 schools from all provinces and territories, with the exception of Prince Edward Island and New Brunswick. [32] Different recruitment strategies were used in the participating Canadian provinces and territories. In each province, a systematic, multi-stage cluster sample approach was used. A list of schools within eligible and consenting boards was created, and classes within schools were randomly sampled from this list. When a school refused participation, the sampling protocol involved selecting a school that was similar as possible to the originally selected school. For the three territories, a census of all students in Grades 6 through 10 was attempted. Due to the sampling methods and coverage, sample weights were developed to promote generalizability of estimates nationally.

**Figure 1:**
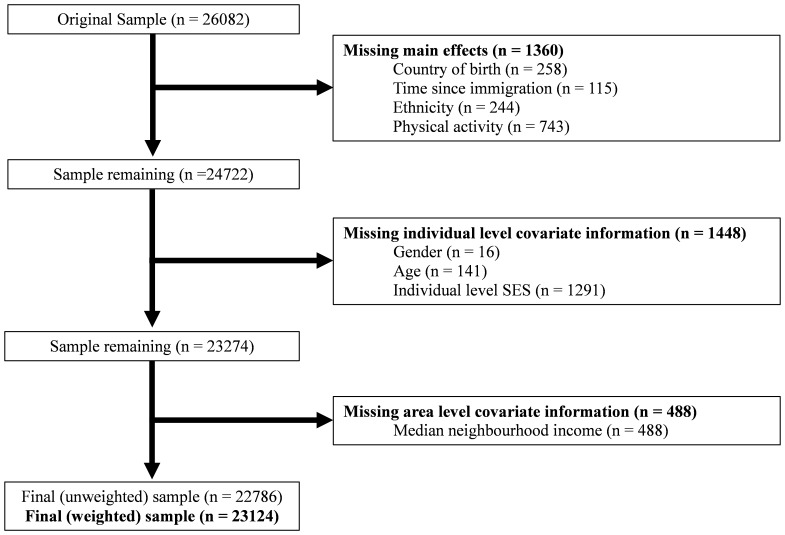
Study flow diagram of exclusion criteria used to eliminate observations from the original HBSC sample.

Approximately 57% of schools approached agreed to participate, and 77% of the estimated students in schools that gave consent participated in the study. Less than 10% declined to participate or spoiled their questionnaires intentionally, and remaining non-participants generally either failed to return consent forms, failed to receive parental consent, or were absent on the day of the survey. [32] From the original sample of 26,078 youth, 3296 were excluded due to missing data (Figure 1). This left a final unweighted sample of 22,786 (weighted sample of 23,124).

Area-level data were obtained from the *2006 Canada Census of Population*. [33] Census-based measures included median neighbourhood income, percentage of immigrants in the community, and Statistics Canada Population Centre Category. [33] Census responses were linked to schools to describe the neighbourhoods in the 1 km radius around each school.

### Ethics Statement

The Canadian HBSC received ethics approval from the Queen’s University General Research Ethics Board (File # GEDUC-430-09). The analyses for this paper received additional ethics approval from the Queen’s University Health Sciences Research Ethics Board (File # 6007744). Consent was sought at multiple levels. First, school jurisdictions were approached for permission to invite their students and schools to participate. Second, school principals were approached to participate. Finally, both active parent consent, in the form of a signed consent form, and passive parent consent, where students were allowed to participate if they did not return the parent consent form indicating their parents refused participation, were used. Participating school jurisdictions and schools selected the consent type that was consistent with local norms, as per ethics agreements at Queen’s University.

### Primary Exposures – Immigrant Generation and Ethnicity

The primary exposure permitted categorization of youth by immigrant generation. This was assessed in the HBSC survey by asking “In which country were you born?” Youth born abroad were categorized as “foreign-born,” while youth born in Canada were classified as “Canadian-born.” Youth were then categorized into five groups by the length of time they have been in Canada by asking “How many years have you lived in Canada?” Response options were: “I was born in Canada,” “1 to 2 years,” “3 to 5 years” “6 to 10 years” and “11 or more years.” Due to small numbers, the last two responses were combined to form a “6+ years” group.

Youth were categorized into seven ethnic groups by asking “How do you describe yourself?” with 16 possible response options. Youth were able to select up to 3 response categories. Responses were used to create the following ethnic groups: “Canadian,” “Arab,” “African,” “South Asian,” “East and South East Asian,” “Latin American” and “Other.” These groups were based on ethnic groupings defined by the 2006 Canadian Census of Population, with three modifications. [33] First, European and North American immigrant youth were combined with Aboriginal youth to create a “Canadian” host culture group (Note: ethics restrictions prohibited separate study of Aboriginal youth). [34] Second, West Asian and South Asian youth were combined due to small numbers of West Asian youth. Finally, an additional group was created (“Other”) that included youth who identified with multiple ethnic groups.

### Outcome – Moderate-to-Vigorous Physical Activity (MVPA)

MVPA was measured by taking an average of the responses to the questions: “Over the past 7 days, on how many days were you physically active for a total of at least 60 minutes per day?” and “Over a typical or usual week, on how many days are you physically active for a total of at least 60 minutes per day?” [35] Prior to being asked these questions, the questionnaire provided students with a description of MVPA and a list of common physical activities of this intensity. MVPA was categorized into three groups of: 0–3 days/week, 4–6 days/week and 7 days/week. Only the highest category (7 days/week) meets Canadian physical activity guidelines. [2], [3] This measure is reasonably valid: Using a 7 day/week cut off, percent agreement is 69.6% between this self-reported measure and objective measures of MVPA determined using accelerometers. [36] This measure was developed using a diverse sample of US adolescents, and has been shown to be appropriate for use in different ethnic groups [35].

### Covariates

Individual-level covariates were age, gender and perceived family wealth, the latter measured through the Family Affluence Scale. [37] These were all found to be important predictors of physical activity among youth. [6], [7], [38], [39] These variables were all obtained via self-report, and measured as part of the 2010 HBSC student questionnaire. [37], [40] School-level covariates were obtained from the 2006 Canadian Census of Population, and were based on a 1 km radius buffer around the school. Previous research using the HBSC survey has found this to be an appropriate buffer by which to make inferences about other social constructs, such as neighbourhood socio-economic status. [41] At the area level, covariates included: Statistics Canada population centre category, percentage of immigrants in the community, and median income quartile [33].

### Analysis Plan

All analyses used a multi-level approach due to the clustered nature of these data and the inclusion of school-level covariates; Level 1 includes individual-level factors and Level 2 includes school-level variables. [42] Cross-tabulations of youth physical activity levels by the exposure categories were performed. All p-values calculated for associated statistical tests used the Rao-Scott chi-square test to control for clustering at the school-level.

Second, etiologic analyses were used to explore the relationship between time since immigration and physical activity using multi-level nominal regressions. Only time since immigration and ethnicity were included in the modelling process, as immigrant generation and time since immigration are collinear. A nominal regression model was built with three outcomes: 0–3 days/week (referent), 4–6 days/week, and 7 days/week of 60 minutes or more of MVPA.

Several models were built, following the approach of Merlo et al. [42] First, an empty model was built. This investigated the random effect of school on the outcome of interest, and explains how much of the variation in MVPA is explained by school alone. [42] Second, a bivariate model was created that only included time since immigration and ethnicity as predictors of physical activity. The third and fourth models controlled for individual-level and school-level covariates respectively. The final model thus included all covariates found to be statistically significant in models three and four. Percentage changes in odds ratios were then calculated for the three iterative models compared to the base model, to determine the impact of these covariates on the effect estimates. Surprisingly, very few covariates created changes in estimates of the primary exposures of greater than 5%. Thus, all variables originally considered were included in the final model.

A third exploratory analysis was conducted that investigated the interaction between time since immigration and ethnicity. This stratified each ethnicity into 4 groups: Canadian-born, 1–2 years, 3–5 years and 6+ years. No variables were controlled for in these models due to insufficient cell sizes.

All analyses were conducted using SAS v9.3 using PROC SURVEYFREQ for cross-tabulations and PROC GLIMMIX for regression models. All analyses considered the sample weights and accounted for clustering at the school-level (SAS Institute, Cary, NC). The intra-class correlation revealed that the school-level accounted for 4.9% of the variation in MVPA, thereby justifying the use of multi-level models [42].

## Results

Our sample was comprised of predominantly Canadian-born youth (90.9%) (Table 1). Most self-identified as Canadian (77.3%). The other major ethnic groups were East and South East Asian (5.8%), African (4.3%), and East Indian and South Asian (3.1%). Only 14.6% of youth reported accumulated 60 minutes of MVPA every day of the week (Table 1), meeting Canadian guidelines and hence were classified as “active”.

**Table 1:** Description of key variables by level of physical activity (n = 23,124).

				Days/week with more than 60 minutes of moderate-to-vigorous physical activity						
		Total		0–3 days/week		4–6 days/week		7 days/week		Rao-Scott Chi-square
		n	% (col)	n	% (row)	n	% (row)	n	% (row)	
**Total n**		23124	100.0	6455	27.9	13282	57.4	3387	14.6	
**Main Effects**										
**Immigrant Generation**	Canadian-born	21025	90.9	5777	27.5	12091	57.5	3157	15.0	Ref
	Foreign-born	2099	9.1	678	32.3	1191	56.7	230	11.0	.0010
**Time Since Immigration**	Canadian-born	21025	90.9	5777	27.5	12091	57.5	3157	15.0	Ref
	6+ years	1134	4.9	345	30.4	663	58.4	126	11.2	.065
	3–5 years	523	2.3	159	30.5	309	59.0	55	10.5	.12
	1–2 years	442	1.9	174	39.3	219	49.6	49	11.1	.0015
**Ethnicity**	Canadian	17883	77.3	4789	26.8	10353	57.9	2741	15.3	Ref
	Arab and West Asian	387	1.7	116	29.9	212	54.8	59	15.2	.66
	African	990	4.3	321	32.4	526	53.1	143	14.4	.065
	East Indian and South Asian	706	3.1	183	25.9	406	57.4	118	16.7	.81
	East and South East Asian	1348	5.8	526	39.0	722	53.5	101	7.5	<.0001
	Latin American	238	1.0	90	37.9	128	53.6	20	8.5	.007
	Other	1571	6.8	430	27.4	937	59.6	204	13.0	.24
**Individual Level Covariates**										
**Gender**	Male	11235	48.6	2580	23.0	6472	57.6	2183	19.4	Ref
	Female	11890	51.4	3875	32.6	6811	57.3	1204	10.1	<.0001
**Perceived family wealth**	Well Off	13171	57.0	3354	25.5	7728	58.7	2089	15.9	Ref
	Average	7762	33.6	2368	30.5	4401	56.7	993	12.8	<.0001
	Worse off	2191	9.5	732	33.4	1153	52.6	306	13.9	<.0001
**Area-Level Covariates**										
**Median Neighbourhood Income**	Quartile 4 (> $67,546)	5366	23.2	1390	25.9	3179	59.2	797	14.8	Ref
	Quartile 3 ($52,650–$67,500)	5361	23.2	1401	26.1	3137	58.5	823	15.3	.94
	Quartile 2 ($43,192–$52,635)	5188	22.4	1572	30.3	2970	57.3	646	12.4	.08
	Quartile 1 (< $43,176)	7210	31.2	2092	29.0	3996	55.4	1122	15.6	.24
**Percentage of Immigrants in Community**	Quartile 4 (>17%)	7868	34.0	2232	28.4	4532	57.6	1104	14.0	Ref
	Quartile 3 (9%–17%)	5992	25.9	1417	23.6	3650	60.9	925	15.4	.009
	Quartile 2 (3%–9%)	5181	22.4	1367	26.4	2931	56.6	883	17.0	.18
	Quartile 1 (<3%)	4083	17.7	1439	35.2	2169	53.1	475	11.6	.0037
**Statistics Canada Population Centre Category**	Large Urban Centre	7637	33.0	2289	30.0	4327	56.7	1021	13.4	Ref
	Medium Centre	4181	18.1	1207	28.9	2379	56.9	595	14.2	.77
	Small Centre	10604	45.9	2817	26.6	6132	57.8	1655	15.6	.08
	Rural	703	3.0	142	20.2	444	63.1	117	16.6	<.0001

Note: N values presented were weighted as per the HBSC protocol. [32] The Rao-Scott chi square test controls for the clustering effect of school. The “other” ethnic group includes youth who identified with more than one of the six identified ethnic groups.

The distribution of the sample across the three physical activity groups implied that foreign-born youth were more physically active than Canadian-born peers (Table 1). After controlling for relevant covariates, youth who immigrated within the last 1–2 years reported being less likely to accumulate at least 60 minutes of MVPA on 4–6 days/week (OR: 0.66 (95% CI: 0.53–0.83)) and 7 days/week (0.62 (0.43–0.89)) in comparison to Canadian born youth (Table 2). Reported MVPA was not significantly different across Canadian born youth and youth who immigrated in the past 3–5 years and 6+ years (Table 2).

**Table 2:** Nominal regression modeling of determinants of moderate-to-vigorous physical activity.

		4–6 days/week				7 days/week			
		Unadjusted		Adjusted		Unadjusted		Adjusted	
Main Effects		OR (95% CI)	p	OR (95% CI)	p	OR (95% CI)	p	OR (95% CI)	p
**Time Since Immigration**	Canadian-born	1.00 (ref)		1.00 (ref)		1.00 (ref)		1.00 (ref)	
	6+ years	1.04 (0.88–1.22)	.67	1.01 (0.86–1.19)	.91	0.85 (0.67–1.10)	.22	0.82 (0.63–1.05)	.12
	3–5 years	0.97 (0.78–1.22)	.81	0.92 (0.73–1.16)	.49	0.89 (0.63–1.26)	.53	0.81 (0.57–1.15)	.24
	1–2 years	0.69 (0.55–0.86)	.001	0.66 (0.53–0.83)	.0003	0.67 (0.47–0.96)	.029	0.62 (0.43–0.89)	.010
**Ethnicity**	Canadian	1.00 (ref)		1.00 (ref)		1.00 (ref)		1.00 (ref)	
	Arab and West Asian	0.87 (0.65–1.15)	.32	0.81 (0.61–1.08)	.15	1.01 (0.68–1.50)	.96	0.93 (0.62–1.39)	.72
	African	0.80 (0.68–0.95)	.012	0.77 (0.65–0.92)	.003	0.86 (0.68–1.10)	.24	0.82 (0.64–1.05)	.12
	East Indian and South Asian	0.97 (0.78–1.21)	.81	0.89 (0.71–1.12)	.32	1.09 (0.80–1.47)	.59	0.97 (0.71–1.32)	.85
	East and South East Asian	0.71 (0.61–0.83)	<.0001	0.67 (0.58–0.79)	<.0001	0.40 (0.31–0.52)	<.0001	0.37 (0.29–0.48)	<.0001
	Latin American	0.80 (0.58–1.11)	.19	0.76 (0.55–1.06)	.10	0.57 (0.34–0.97)	.037	0.53 (0.31–0.91)	.022
	Other	0.99 (0.88–1.12)	.91	1.00 (0.89–1.12)	.97	0.88 (0.74–1.04)	.13	0.90 (0.76–1.07)	.24

Note: The reference group in both models was “0–3 days/week.” Both models (unadjusted and adjusted) control for clustering at the school level. The adjusted models control at the individual level for age, gender and perceived family wealth. At the area level the models control for median neighbourhood income, percentage of immigrants in the community and Statistics Canada Population Centre category.

Differences in MVPA were also observed by ethnicity. East and South East Asian, Latin American, and African ethnic groups had lower physical activity levels, with 32–39% accumulating the recommended 60 minutes of MVPA on only 0–3 days/week, compared to 26.8% within the Canadian ethic group (Table 1). These differences persisted after controlling for important covariates (Table 2).

Exploratory analyses tested the potential interaction between ethnicity and time since immigration (Table 3). Tests for interaction were not statistically significant (p = .12). Irrespective of their time since immigration, East/South East Asian youth were less active than Canadian born youth who identified as Canadian. For other ethnicities no trends emerged, although different relationships were seen with increased time since immigration.

**Table 3:** Investigations of interactions between ethnicity and time since immigration.

		Total		0–3 days/week		4–6 days/week		7 days/week		Rao-Scott Chi-square
Ethnicity	Time since Immigration	n	% (col)	n	% (row)	n	% (row)	n	% (row)	
Canadian	Canadian-born	17358	99.5	4655	26.8	10028	57.8	2675	15.4	Ref
	6+ years	332	35.2	85	25.7	199	60.1	47	14.2	.85
	3–5 years	105	11.2	21	19.5	71	67.9	13	12.6	.20
	1–2 years	88	0.5	28	32.0	54	61.4	6	6.6	.15
Arab and West Asian	Canadian-born	228	82.4	69	30.3	119	51.9	40	17.7	.53
	6+ years	103	27.5	25	24.0	71	69.6	7	6.4	.06
	3–5 years	42	10.7	15	36.7	17	39.5	10	23.8	.19
	1–2 years	14	2.9	7	46.3	6	39.6	2	14.1	.44
African	Canadian-born	740	83.3	234	31.6	393	53.1	113	15.3	.17
	6+ years	123	13.4	41	33.1	69	56.2	13	10.7	.37
	3–5 years	55	4.6	14	25.6	36	64.2	6	10.2	.68
	1–2 years	71	6.3	32	45.4	28	39.7	11	14.8	.087
East Indian and South Asian	Canadian-born	423	64.6	104	24.7	240	56.8	78	18.5	.47
	6+ years	135	20.7	41	30.3	77	56.6	18	13.2	.87
	3–5 years	96	14.7	23	24.0	64	66.5	9	9.5	.40
	1–2 years	52	7.9	14	27.8	25	47.5	13	24.8	.36
East and South East Asian	Canadian-born	749	79.5	287	38.3	404	54.0	58	7.7	<.0001
	6+ years	279	63.5	99	35.7	157	56.5	22	7.8	.011
	3–5 years	160	36.5	63	39.1	86	53.6	12	7.3	.005
	1–2 years	160	36.5	77	47.7	74	46.1	10	6.2	<.0001
Latin American	Canadian-born	128	50.2	44	34.2	73	57.0	11	8.8	.12
	6+ years	61	28.2	29	46.7	29	46.6	4	6.7	.023
	3–5 years	28	9.7	13	46.7	11	40.3	4	13.0	.20
	1–2 years	21	4.2	5	23.2	15	71.6	1	5.3	.38
Other	Canadian-born	1410	96.2	391	27.7	838	59.4	181	12.9	.19
	6+ years	95	6.2	20	21.2	60	63.3	15	15.6	.64
	3–5 years	33	2.2	8	24.5	24	70.6	2	4.9	.39
	1–2 years	33	1.9	11	33.1	15	46.7	7	20.2	.69

Note: N values presented were weighted as per the HBSC protocol. [32] The Rao-Scott chi square test controls for the clustering effect of school.

## Discussion

The most important findings of the study of immigrant generation, ethnicity and physical activity among young people were as follows. First, immigrant youth in Canada are less active than their Canadian-born peers. Second, reported physical activity increases with increased time since immigration. Third, reported physical activity differs by ethnicity. Finally, exploratory tests of possible interactions between immigrant generation and ethnicity were generally negative, but do suggest that East and South East Asian youth have reduced physical activity levels irrespective of their immigration status and the length for which they have resided in Canada.

Our primary finding that the observed reduction in physical activity levels in immigrant youth decreased as time since immigration increased supports the theory of acculturation. That is, as immigrants live longer in a country, their physical activity behaviours more closely approximate those of the host culture. This finding is consistent with research findings for Canadian adults. Cross-sectional analyses of 400,055 adult participants in the Canadian Community Health Survey (2000–2005) indicate that recent immigrants (<10 years) are 2.68 (95% CI: 2.54–2.83) times more likely to get no meaningful physical activity compared to non-immigrants whereas established immigrants (>10 years) are only marginally more likely to get no meaningful physical activity (OR: 1.30, 95% CI: 1.26–1.35). [27] Our findings echo these results, except that they showed that immigrant youth match the physical activity behaviours of their Canadian-born peers within a few years. Findings of similar size and direction have been reported among a national sample of US youth. Foreign born youth had 1.39-fold (95% CI: 1.13–1.70) increased odds of obtaining no physical activity compared to US-born peers of US-born parents, i.e. 2^nd^ (and higher) generation youth. [25] Studies of Swedish youth found similar findings, with foreign-born youth being less physical active in sports than Swedish born peers (54.4% vs 41.1%, p = .003) [43] Our results suggest these findings may be driven by certain ethnic groups, and are not generalizable to all immigrant youth.

We found that East and South East Asian youth had drastically reduced odds of being physical active regardless of time since immigration. These findings and the magnitude of these associations are supported by other Canadian studies. [30], [31] A study of youth in Montreal found increased odds of no physical activity of similar effect sizes to our study, with Asian boys and girls reporting odds of 2.1 (1.4–3.1) and 1.8 (1.2–2.6), respectively, for no physical activity. [30] This difference remains consistent regardless of time since immigration. A study of Canadian adults reported slightly smaller effect sizes compared to our study, with established (>10 years) and recent (<10 years) East and South East Asian immigrant adults reporting odds of 0.6 (0.5–0.8) and 0.7 (0.6–0.9) for moderate-to-high physical activity, compared to White immigrants within the same time since immigration category. [31] These differences between East and South East Asian immigrants and Canadian adult peers may be due to 1) being involved in different forms of physical activity, 2) cultural differences in what constitutes physical activity, and 3) ethnic differences in extracurricular activity involvement. [27], [44], [45] Studies of youth in the US have reported similar findings, although they focused on Hispanic and non-Hispanic Black youth. In both cases, they found that foreign born youth with both parents born abroad had increased odds of obtained no physical activity (OR: 2.13 (95% CI: 1.67–2.71) and 1.46 (95% CI: 0.88–2.41) respectively). [25] Studies in the US have focused on Korean and South Asian adults, and have reported similar findings, corroborating what we saw in our study among East and South East Asian youth. [46], [47] Finally, studies in Europe have report ethnic minority adolescents are less active than Norwegian peers [48].

These findings reinforce the potential importance of tailoring physical activity immigrant-specific interventions by ethnicity, and including those born in Canada. A systematic review of studies of youth have found that multi-component interventions, incorporating the school environment, family and child have been most effective. [49] This has been shown among interventions performed in the US, [50] as well as similar programs in Europe. [51]–[53] Previous interventions have focused on youth as a homogenous group, and our findings support the hypothesis that ethnic differences exist within youth, and specifically the immigrant youth population. These differences have been shown to be barriers to participation in physical activity. [47] Thus acknowledging cultural norms and values as part of interventions may further their effectiveness.

These findings have significant implications for health promotion efforts in Canada. A large proportion (19.8%) of Canadians were born abroad. [33] One potential mechanism by which immigrant youth may be less active than Canadian-born peers is due to participation in sports. While 55% of children of Canadian-born parents participated in sports, only 32% of children of parents who immigrated to Canada in the last 10 years participate. [38] A second important implication is that a large number of European, Canadian and US immigrants are from Asia and the Pacific. [18]–[20] Our finding that these youth have drastically lower odds of obtaining sufficient MVPA, a finding that is supported by national studies of Canadian adults, suggests that these individuals may have lower physical activity levels throughout the life course. [29], [31] The potential reasons for these differences demand further study and suggest the need for tailored and culturally sensitive interventions.

The main strength of this study is its novelty within the physical activity literature. Methodologically, we controlled for the potential confounding effects of both area and individual level factors. In addition, this study investigated both the main effects of ethnicity and time since immigration on physical activity, as well as the interaction between the two. However, this study also has several limitations. First, since this is a cross-sectional study, we cannot confirm the temporal sequence of the observed associations. Second, we cannot determine the levels of physical activity the youth may have had in their heritage country. Third, the individual-level measures were all obtained via self-report, although they have demonstrated reliability and validity. [35], [37], [40] In addition, this analysis could not compare 1^st^, 2^nd^ and 3^rd^ generation youth, as there was no information available on parents’ country of birth, and this has been shown to be associated with child sport involvement in Canada. [38] Finally, we were unable to investigate Aboriginal youth separately from our “Canadian host culture” group, due to ethics restrictions. This is a group with unique health behaviours, attitudes and culture.

## Conclusion

This study indicates that both immigration and ethnicity play important roles as determinants of physical activity in populations of young people. Future research should investigate mechanisms by which ethnic groups differ in physical activity levels, as these differences will provide tangible areas for interventions. These studies could include longitudinal analyses of specific ethnic groups to determine factors that change following immigration, mixed-methods or qualitative research with specific groups to uncover mechanisms responsible for these changes, or parent-child studies that investigate the role of the parent in child physical activity levels following immigration. For public health professionals, our findings suggest that creating ethnicity-specific interventions may be important, especially for the East and South East Asian population in Canada. These interventions can focus on both increasing activity and reducing sedentary behaviours, but require evidence to ensure they are effective. It is important to determine how best to encourage these youth to adopt healthy lifestyles and behaviours, as these behaviours may stay with them through the life course.
